# Identification of Mutations Conferring Tryptanthrin Resistance to *Mycobacterium smegmatis*

**DOI:** 10.3390/antibiotics10010006

**Published:** 2020-12-23

**Authors:** Svetlana G. Frolova, Ksenia M. Klimina, Ravinder Kumar, Aleksey A. Vatlin, Deepak B. Salunke, Pravin Kendrekar, Valery N. Danilenko, Dmitry A. Maslov

**Affiliations:** 1 Laboratory of Bacterial Genetics, Vavilov Institute of General Genetics, Russian Academy of Sciences, Moscow 119333, Russia; sveta.frolova.1997@bk.ru (S.G.F.); ppp843@yandex.ru (K.M.K.); vatlin_alexey123@mail.ru (A.A.V.); valerid@vigg.ru (V.N.D.); 2 Moscow Institute of Physics and Technology (State University), Dolgoprudny 141701, Russia; 3 Department of Molecular Biology and Genetics, Federal Research and Clinical Center of Physical-Chemical Medicine of Federal Medical Biological Agency, Moscow 119435, Russia; 4 Department of Chemistry and Centre of Advanced Studies in Chemistry, Panjab University, Chandigarh 160014, India; binderdhull@gmail.com (R.K.); salunke@pu.ac.in (D.B.S.); 5 Department of Design of Functional Foods and Nutritionology, Moscow State University of Food Production, Moscow 109316, Russia; 6 National Interdisciplinary Centre of Vaccine, Immunotherapeutics and Antimicrobials, Panjab University, Chandigarh 160014, India; 7 Unit for Drug Discovery Research (UDDR), Department of Health Sciences, Central University of Technology, Bloemfontein 9300, South Africa; kkpravin@gmail.com

**Keywords:** tuberculosis, mycobacterium smegmatis, tryptanthrin, drug resistance, efflux

## Abstract

Tuberculosis (TB), caused by *Mycobacterium tuberculosis*, is a global burden, responsible for over 1 million deaths annually. The emergence and spread of drug-resistant *M. tuberculosis* strains (MDR-, XDR- and TDR-TB) is the main challenge in global TB-control, requiring the development of novel drugs acting on new biotargets, thus able to overcome the drug-resistance. Tryptanthrin is a natural alkaloid, with great therapeutic potential due to its simple way of synthesis and wide spectrum of biological activities including high bactericidal activity on both drug-susceptible and MDR *M. tuberculosis* strains. InhA was suggested as the target of tryptanthrins by in silico modeling, making it a promising alternative to isoniazid, able to overcome drug resistance provided by *katG* mutations. However, neither the mechanism of action of tryptanthrin nor the mechanism of resistance to tryptanthrins was ever confirmed in vitro. We show that the MmpS5-MmpL5 efflux system is able to provide resistance to tryptanthrins using an in-house test-system. Comparative genomic analysis of spontaneous tryptanthrin-resistant *M. smegmatis* mutants showed that mutations in *MSMEG_1963* (EmbR transcriptional regulator) lead to a high-level resistance, while those in *MSMEG_5597* (TetR transcriptional regulator) to a low-level one. Mutations in an MFS transporter gene (*MSMEG_4427*) were also observed, which might be involved in providing a basal level of tryptanthrins-resistance.

## 1. Introduction

*Mycobacterium tuberculosis* is the causative agent of tuberculosis (TB), one of the most dangerous infectious diseases. According to the World Health Organization, about 10 million people fell ill with TB and more than 1.4 million people died in 2019, which makes TB one of the top 10 causes of death worldwide, with an overall number of TB-infected people reaching 2 billion. The global TB incidence rate has fallen by 9% between 2015 and 2020, though it failed to reach the goal of a 20% reduction stated by the WHO TB end strategy, with the COVID-19 pandemic becoming one of the possible reasons affecting further progress of this goal. Drug-resistant TB is a serious obstacle to a successful cure and requiring prolonged and expensive treatment. Globally, in 2019, 3.3% of new TB cases and 17.7% of previously treated TB cases had multidrug-resistance (MDR-TB, defined as TB resistant to rifampicin and isoniazid) [1]. *M. tuberculosis* drug resistance is usually defined by mutations in genes encoding drug biotargets, activators of pro-drugs, and efflux pumps’ transcriptional regulators [2].

The emergence and spread of *M. tuberculosis* strains with extensive (XDR-TB, defined as MDR with additional resistance to second-line injectables and a fluoroquinolone) and total drug resistance (TDR-TB, defined as TB resistant to all anti-TB drugs) urge for new approaches in design and the screening of novel antitubercular drugs [3,4,5,6].

**Scheme 1:**
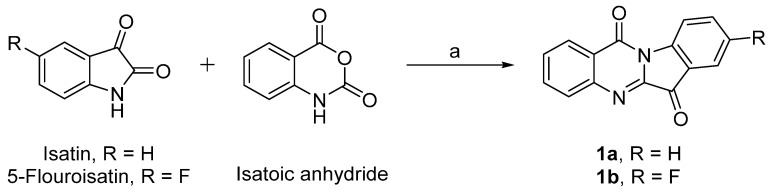
Synthetic route for the preparation of tryptanthrins. Reagents and conditions: (a) triethylamine, toluene, 110 °C, 4 h, 90%.

Tryptanthrin (indolo[2,1-*b*]quinazoline-6,12-dione) is a natural alkaloid compound, belonging to indoloquinazoline antibiotics, and was first isolated from a Chinese herb *Strobilanthescusia Kuntze* [7]. Tryptanthrin (**1a**, Scheme 1) has great therapeutic potential due to its simple way of synthesis and wide spectrum of biological activities such as antifungal, antibacterial, and antiprotozoal [7,8,9]. Tryptanthrin and its analogs were shown to be anti-tumor agents both in vitro on cancer cell lines [10,11] and in vivo on rat model [12], being potent inhibitors of indoleamine 2,3-dioxygenase (IDO1) [13], tryptophan 2,3-dioxygenase (TDO) and indoleamine 2,3-dioxygenase 2 (IDO2) [14]. The anti-inflammatory action of tryptanthrin was shown to be effective for treating intestine disorders in murine models [15,16] and inhibiting allergic responses in vitro [17].

Tryptanthrin was first described as a potential anti-TB agent by Mitscher and Baker, with a MIC of 1 µg/mL, preserving its activity on MDR strains [18]. According to docking studies, tryptanthrin exhibits a high affinity towards *M. tuberculosis* enoyl-acyl carrier protein reductase (InhA) [19]—an enzyme playing a key role in the biosynthesis of mycolic acids, a primary target of isoniazid [20]. Isoniazid is a prodrug, that needs to be activated by the catalase-peroxidase encoded by *katG* [21], and the majority of drug-resistance conferring mutations occur in *katG* rather than *inhA* [22]. Thus, tryptanthrins, which do not need to be activated by katG, could represent promising anti-TB drugs, that could overcome *katG*-mediated drug resistance to isoniazid. Despite the in silico studies showing that tryptanthrins target InhA, the exact biotarget of these compounds, as well as the mycobacterial resistance mechanism, have not yet been described in vitro.

This paper describes the mechanisms of mycobacterial resistance to tryptanthrins by evaluating the biological activity of tryptanthrin and its fluoro-substituted derivative (8-Fluorotryptanthrin, **1b**) [19,23] on *Mycobacterium smegmatis* strains, obtaining their spontaneous drug-resistant mutants, their whole-genomic sequencing, and comparative genomic analysis.

## 2. Results

### 2.1. Synthesis of Tryptanthrin Analogs

Isatin and 5-fluoroisatin (Scheme 1) on condensation with isatoic anhydride in the presence of triethylamine in refluxing toluene for four hours resulted in the formation of tryptanthrin (**1a**) and 8-fluorotryptanthrin (**1b**), respectively [24]. The formation of the desired compounds was confirmed using NMR spectroscopic analysis and the purity was analyzed using HPLC (Figures S1–S5).

### 2.2. Tryptanthrins May Be Subjected to MmpS5-MmpL5 Mediated Efflux

The MmpS5-MmpL5 efflux system was previously shown to provide resistance to multiple drugs in different mycobacterial species [25,26,27], including resistance to bedaquiline in clinical *M. tuberculosis* isolates [28]. We determined the minimal inhibitory concentrations (MICs) of **1a** and **1b** on three *M. smegmatis* strains differing in *mmpS5-mmL5* operon expression levels: *mc2 155*, *Δmmp5* and *atr9c*. *M. smegmatis Δmmp5* is an *M. smegmatis mc2 155* derivative, carrying a 2828 bp. deletion in the *mmpS5-mmpL5* operon, and hypersensitive to drugs, subjected to MmpS5-MmpL5 efflux, such as imidazo[1,2-*b*][1,2,4,5]tetrazines [29]. *M. smegmatis atr9c,* on the contrary, carries a mutation in *MSMEG_1380*, leading to overexpression of *mmpS5-mmpL5* genes, and is resistant to drugs, subjected to MmpS5-MmpL5 efflux [27]. Thus, comparing MIC values on these three strains can be used for prescreening antimycobacterial drugs candidates for potential MmpS5-MmpL5 efflux mediated drug resistance.

*M. smegmatis atr9c* turned out to be resistant to compound **1a**, while *M. smegmatis Δmmp5* was hypersensitive to **1b** (Table 1), showing that MmpS5-MmpL5 efflux was involved in resistance to tryptanthrins, but with different specificity.

### 2.3. Mutations in Transcriptional Regulators and a Transporter Gene May Confer Resistance to Tryptanthrins

As *mmpS5-mmpL5* overexpression led to a relatively high level of resistance to tryptanthrin (**1a**), other spontaneous mutations in *MSMEG_1380* could also result in **1a** resistance. Thus, we selected **1b** for further generation of spontaneous drug-resistant mutants. *M. smegmatis Δmmp5* was used as the parent strain together with *M. smegmatis mc2 155*, as it could also help to avoid *mmpS5-mmpL5*-mediated drug resistance, and to reveal other resistance-conferring mutations.

We were able to obtain spontaneous **1b**-resistant mutants derived from both *M. smegmatis mc2 155* and *M. smegmatis Δmmp5* at a frequency of 6.3 × 10^−7^ and 1.6 × 10^−7^, respectively. We randomly selected eight mutants (four from each group) for further analysis.

Whole-genomic sequencing and comparative genomic analysis allowed us to identify a number of non-synonymous mutations, which may confer tryptanthrin-resistance to *M. smegmatis* (Table 2).

We identified three nonsynonymous single nucleotide polymorphisms (SNPs) in four strains in *MSMEG_1963*, encoding an EmbR transcriptional regulatory protein, leading to a high-level resistance to both tryptanthrins (Table 2). Interestingly, this protein’s homolog in *M. tuberculosis* (Rv1267c) was shown to be involved in ethambutol resistance [30]. However, the MIC values of ethambutol were revealed to be the same on the strains carrying *MSMEG_1963* mutation and the control strains (0.1 µg/mL), showing no cross-resistance.

The *MSMEG_5597* had three different mutations in four strains: one frameshift mutation, one SNP, leading to prolonged protein synthesis, and an insertion of ISMsm1 transposon (genes *MSMEG_1728-MSMEG_1730*). These mutations were associated with low-level resistance to tryptanthrins (Table 2).

We also identified one SNP in all the *M. smegmatis mc2 155* derived mutants in *MSMEG_4427,* encoding a Major Facilitator Superfamily (MFS) transporter. BLAST search identified Rv1250 as its homolog in *M. tuberculosis* with a 32% identity and 46% similarity in the amino acid sequence. *Rv1250* was reported to be overexpressed with other efflux pump genes in MDR *M. tuberculosis* strains [31,32,33,34] and can be upregulated in the presence of drugs [35]. Besides the effect of overexpression of *Rv1250* on drug resistance, unique SNPs in this gene were also found in XDR-TB strains [36].

## 3. Discussion

Tryptanthrins attracted attention as potential anti-tuberculosis drug candidates due to their high in vitro activity against both drug-susceptible and MDR *M. tuberculosis* strains, and their low toxicity [18,23,37]. InhA was suggested as the target of tryptanthrins by in silico modeling [19], making it a promising alternative to isoniazid, able to overcome drug resistance provided by *katG* mutations [22]. However, neither the mechanism of action nor the mechanism of resistance to tryptanthrins was studied in vitro, which is an essential step of drug development in the post-genomic era [38]. We describe several potential ways of *M. smegmatis* developing resistance to tryptanthrins.

MmpS5-MmpL5 is an important multidrug-efflux-pump system, which is able to provide mycobacteria resistance to multiple drugs such as bedaquiline, clofazimine [39], azoles [25], thiacetazone derivatives [26], and imidazo[1,2-*b*][1,2,4,5]tetrazines [27]. We used a test-system involving two *M. smegmatis* recombinant strains to show that *mmpS5-mmpL5* operon overexpression provides resistance to tryptanthrin (**1a**), while deletion of *mmpS5-mmpL5* operon leads to hypersensitivity to 8-fluorotryptanthrin (**1b**). Compound **1a** thus may be subjected to MmpS5-MmpL5-mediated efflux more efficiently, as *mmpS5*-*mmpL5* overexpression leads to resistance to this compound. However, the MmpS5-MmpL5 is able to provide a basal level of resistance to the compound **1b** as well, though it is not increased with this systems’ overexpression. Apparently, the basal level of resistance to **1b** is more specifically mediated by MmpS5-MmpL5 efflux, while the basal level of resistance to **1a** may be less specific and provided by other efflux pumps as well. Thus, the MmpS5-MmpL5 system was able to provide resistance to both tryptanthrins (though at different levels), and it should be considered when developing other tryptanthrin derivatives, as potentially leading to cross-resistance with other drugs.

Whole-genomic sequences of spontaneous *M. smegmatis* mutants, obtained on **1b**, but resistant to both compounds revealed mutations in three genes, which may be a clue for drug-resistance or action mechanism.

The mutation in a transcriptional regulator (*MSMEG_1963*) led to a high level of resistance to both compounds in the strains where it was present. The homolog of this gene in *M. tuberculosis* is described as the transcriptional regulator of the *embCAB* operon, with mutations in it leading to ethambutol resistance [30]. However, the lack of cross-resistance with ethambutol in *M. smegmatis* may show a different function of this gene.

Mutations found in *MSMEG_5597* (frameshift, insertion of a transposon, and a longer protein product), associated with low-level resistance to tryptanthrins, apparently lead to a disruption of its function. *MSMEG_1730* encodes a TetR family transcriptional regulator. TetR family proteins are involved in the transcriptional control of multidrug efflux pumps, pathways for the biosynthesis of antibiotics, response to osmotic stress and toxic chemicals, control of catabolic pathways, differentiation processes, and pathogenicity [40]. Disruption of a TetR repressor’s function usually leads to the upregulation of some other genes. However, this gene does not have any efflux pump genes located nearby on the chromosome. The *MSMEG_5596* located upstream of *MSMEG_5597,* which may be regulated by the latter, encodes an SDR family oxidoreductase, which might be involved in tryptanthrin modification, though this requires additional investigation.

While the mutation in a transcriptional regulator most likely leads to a change in expression levels of its regulated genes, the mutation found in the *MSMEG_4427* encoding MFS transporter may be directly altering its specificity to enhance tryptanthrins’ efflux or alter cell wall permeability. This kind of transporters is responsible for the efflux of a wide spectrum of compounds with different chemical and physical properties. MFS transporters use the energy stored in an electrochemical gradient across the membrane [41]. Importantly, MFS transporters play a significant role in bacterial intrinsic drug resistance [42]. Though in most cases this type of transporters in *M. tuberculosis* was reported to confer drug resistance by their overexpression [31,32,33,34], SNPs associated with XDR were also reported for *Rv1250* (homolog of *MSMEG_4427*) [36]. The strains carrying the mutation in *MSMEG_4427* showed a higher level of resistance to tryptanthrins, when combined with the *MSMEG_5597* mutation (*mc2-8*, *mc2-16* and *mc2-18* compared to *Δmmp5-10*). However, the *mc2*-strains also had a functional copy of *mmpS5-mmpL5* operon, able to provide a higher basal level of resistance. Thus, the impact of *MSMEG_4427* mutation on tryptanthrins resistance is less evident than of those in *MSMEG_1963* and *MSMEG_5597.*

Though the individual impact of each mutation on tryptanthrins resistance is yet to be established by overexpression studies and targeted mutants’ construction, we believe that the results presented in this paper can be a clue to further investigation of tryptanthrins’ mechanism of action and mycobacterial resistance to these compounds. Other challenges in tryptanthrins’ development as anti-tuberculosis drug candidates include the design of more soluble derivatives with higher in vivo potencies [43].

## 4. Materials and Methods

### 4.1. Synthetic Procedures of Tryptanthrin Analogs

Indolo[2,1-*b*]quinazoline-6,12-dione (Tryptanthrin, **1a**): Isatin (1 g, 6.796 mmol, 1 eq) was dissolved in toluene (20 mL). Et_3_N (4.74 mL, 33.98 mmol, 5 eq) was added and the reaction mixture was stirred for 15 min. Isatoic anhydride (1.66 g, 10.19 mmol, 1.5 eq) was added to the reaction mixture and the resulting mixture was refluxed at 110 °C for 4 h. Toluene was evaporated under reduced pressure and the product obtained was extracted in ethyl acetate. The ethyl acetate layer was washed with water followed by brine and dried over anhydrous Na_2_SO_4_ and concentrated under vacuum to yield a crude yellow product which was subjected to a flash column chromatography to isolate pure product (eluent: 15% ethyl acetate in hexanes). Yellow solid, R*_f_* = 0.35 (30% Ethyl acetate/Hexanes) ^1^H NMR (400 MHz, CDCl_3_) δ 8.63 (d, *J* = 8.1 Hz, 1H), 8.44 (dd, *J* = 7.9, 1.2 Hz, 1H), 8.03 (dd, *J* = 8.1, 0.7 Hz, 1H), 7.92 (dd, *J* = 7.6, 0.7 Hz, 1H), 7.89–7.83 (m, 1H), 7.79 (td, *J* = 8.0, 1.3 Hz, 1H), 7.71–7.65 (m, 1H), 7.43 (td, *J* = 7.6, 0.8 Hz, 1H). HPLC Purity- 98.20%.

8-Fluoro-indolo[2,1-*b*]quinazoline-6,12-dione (8-Fluorotryptanthrin, **1b**): Compound **1b** was synthesized following the same procedure as described for **1a** using 5-flouroisatin. The synthesis and purification were optimized for 1 g scale. Yellow solid, R*_f_* = 0.3 (30% Ethyl acetate/Hexanes); ^1^H NMR (500 MHz, CDCl_3_) δ 8.58 (dd, *J* = 8.7, 4.0 Hz, 1H), 8.35 (d, *J* = 7.8 Hz, 1H), 7.95 (d, *J* = 8.0 Hz, 1H), 7.79 (t, *J* = 7.6 Hz, 1H), 7.61 (t, *J* = 7.5 Hz, 1H), 7.51 (dd, *J* = 6.4, 2.5 Hz, 1H), 7.41 (td, *J* = 8.6, 2.6 Hz, 1H); ^13^C NMR (126 MHz, CDCl_3_) δ 181.7, 162.1, 157.9, 146.5, 135.3, 130.9, 130.5, 127.6, 124.9, 124.8, 123.7, 119.7, 119.7, 112.2, 112.0. HPLC Purity- 99.04%.

### 4.2. Bacterial Strains and Growth Conditions

All *M. smegmatis* strains were grown in Middlebrook 7H9 medium (Himedia, Mumbai, India) supplemented with oleic albumin dextrose catalase (OADC, Himedia, Mumbai, India), 0.1% Tween-80 (*v*/*v*), and 0.4% glycerol (*v*/*v*) or on solid medium M290 Soyabean Casein Digest Agar (Himedia, Mumbai, India). Bacterial cultures in liquid medium were incubated in the Multitron incubator shaker (Infors HT, Basel, Switzerland) at 37 °C and 250 rpm.

### 4.3. MIC Determination

Minimal inhibitory concentrations (MICs) of the studied compounds on *M. smegmatis* were determined in a liquid medium. *M. smegmatis* strains were cultured overnight in 7H9 medium, then diluted in the proportion of 1:200 in fresh medium (to approximately OD_600_ = 0.05). 196 µL of the diluted culture were poured in sterile non-treated 96-well flat-bottom culture plates (Eppendorf, Hamburg, Germany) and 4 µL of serial two-fold dilutions of the tested compounds in DMSO were added to the wells to reach final concentrations ranging from 0.5 to 32 µg/mL, the maximum soluble concentrations of **1a** and **1b** were also tested (49.3 and 53.2 µg/mL respectively, equivalent to 0.2 mM). For ethambutol MIC determination, the serial two-fold dilutions ranged from 0.05 to 6.4 µg/mL. The plates were incubated at 37 °C and 250 rpm for 48 h. The MIC was determined as the lowest concentration of the compound with no visible bacterial growth.

### 4.4. Generation of Resistant Mutants and Their Phenotype Characterization

*M. smegmatis mc2 155* and *M. smegmatis Δmmp5* were grown in a liquid medium to reach OD_600_ = 2.8 (~4 × 10^8^ CFU/mL). 200 µL of each bacterial culture were plated on agar plates containing **1b** at a final concentration of 16 µg/mL (4× MIC for *M. smegmatis mc2 155* and 16× MIC *M. smegmatis Δmmp5*). Plates were incubated in a thermostat at 30 °C for three days until the emergence of single colonies. The colonies from two plates for each strain were counted to determine the frequency of drug-resistant mutants’ emergence. Serial 10-fold dilutions of each bacterial culture were plated on compound-free plates to determine the exact titer. The mutants’ resistance phenotypes were confirmed by streaking several colonies on M290 plates containing 16 µg/mL of **1b**. The parental strains (*M. smegmatis mc2 155* and *Δmmp5*) were used as control. Tryptanthrins’ MICs on resistant mutants were determined in a liquid medium as described above.

### 4.5. Mycobacterial DNA Extraction

Mycobacterial genomic DNA was isolated from 10 mL by enzymatic lysis as described by Belisle et al. [44], after preliminary isolation, DNA was treated with RNase A (Thermo Fisher Scientific, Waltham, MA, USA) and extracted in the phenol-chloroform-isoamyl alcohol solution (25:24:1).

### 4.6. Whole-Genomic Sequencing

A total of 250 ng genomic DNA was taken for shotgun sequencing library preparation. After DNA sonication on Covaris S220 System (Covaris, Woburn, MA, USA), the size (400–500 bp) and quality of fragmented samples were assessed on Agilent 2100 Bioanalyzer (Agilent, Santa Clara, CA, USA) according to the manufacturer’s manual. NEBNext Ultra II DNA Library Prep Kit (New England Biolabs, Ipswich, MA, USA) was used for pair-ended library preparation, and NEBNext Multiplex Oligos kit for Illumina (96 Index Primers, New England Biolabs, Ipswich, MA, USA) was used for libraries’ indexing. The libraries were quantified by Quant-iT DNA Assay Kit, High Sensitivity (Thermo Scientific, Waltham, MA, USA). DNA sequencing (2 × 125 bp) was performed on the HiSeq 2500 platform (Illumina, San Diego, CA, USA) according to the manufacturer’s recommendations.

### 4.7. Whole-Genomic Data Analysis

The obtained reads’ quality was assessed with FastQC (v. 0.11.9) [45], which revealed good quality for further assembly. The reads were aligned to the reference genome (NC_008596.1, PRJNA57701) using the BWA-MEM algorithm [46]. The pileup was generated by mpileup (-B -f) in SAMtools [47]. Single nucleotide variants were called by running mpileup2snp (--min-avg-qual 30 --min-var-freq 0.80 --*p*-value 0.01 --output-vcf 1 --variants 1) in VarScan (v. 2.3.9) [48]. Annotation was created using vcf_annotate.pl (developed by Natalya Mikheecheva of the Laboratory of Bacterial Genetics, Vavilov Institute of General Genetics). The non-synonymous single nucleotide variants found within open reading frames and absent in the wild-type strain were selected for further analysis.

For de novo assembly, reads were trimmed with Trimmomativ (v. 0.39) [49] with the following settings, as recommended in SPAdes manual: ILLUMINACLIP:NexteraPE-PE.fa:2:30:10 LEADING:3 TRAILING:3 SLIDINGWINDOW:4:20 MINLEN:36. SPAdes (v. 3.14.1) [50] with --isolate setting was used for de novo assembly. BLAST (https://blast.ncbi.nlm.nih.gov) was used to detect the transposon insert in *M. smegmatis mc2-16* and *mc2-18* genomes, as well as for the homology search.

### 4.8. Data Availability

The raw sequencing data (SRA), as well as the WGS data for *M. smegmatis* strains obtained in this study, are publicly available in NCBI GenBank (BioProject ID: PRJNA672137).

## 5. Conclusions

Tryptanthrins are considered promising antituberculosis agents, active on both drug-susceptible and MDR-TB strains, with InhA predicted as their primary target. In this research we show that MmpS5-MmpL5-mediated efflux can provide mycobacteria resistance to tryptanthrins and may thus lead to cross-resistance with other drugs. We also revealed mutations in *MSMEG_1963* (EmbR transcriptional regulator) as providing high-level resistance to tryptanthrins, and those in *MSMEG_5597* (TetR transcriptional regulator) as leading to a low-level one. Mutations in an MFS transporter gene (*MSMEG_4427*) might be involved in providing a basal level of tryptanthrins-resistance.

## Figures and Tables

**Table 1:** MICs of **1a** and **1b** on *M. smegmatis* strains.

Compound	*M. smegmatis* Strains MICs						
	Mw	*mc2 155* ^1^		Δ*mmp5* ^2^		*atr9c* ^3^	
		µg/mL	µM	µg/mL	µM	µg/mL	µM
**1a**	248.24	4	16.1	4	16.1	16	64.4
**1b**	266.23	4	15	1	3.8	4	15

^1^ Wild type strain. ^2^ Strain lacking *mmpS5-mmpL5* operon. ^3^ Strain with *mmpS5-mmpL5* operon overexpression.

**Table 2:** Genetic and phenotypic characteristics of *M. smegmatis* mutant strains.

Strain	Gene						MICs, µg/mL	
	*MSMEG_1963*		*MSMEG_5597*		*MSMEG_4427*		1a	1b
	Nucleotide	Amino Acid	Nucleotide	Amino Acid	Nucleotide	Amino Acid		
*mc2-8*	w.t.	w.t.	C49>CTG	frameshift	CTG>CGG	L236R	16	16
*mc2-10*	CGT>TGT	R893C	w.t.	w.t.	CTG>CGG	L236R	>49	>53
*mc2-16*	w.t.	w.t.	Transposon insertion		CTG>CGG	L236R	16	8
*mc2-18*	w.t.	w.t.	Transposon insertion		CTG>CGG	L236R	32	8
Δ*mmp5-1*	CGC>TGC	R523C	w.t.	w.t.	w.t.	w.t.	>49	>53
Δ*mmp5-3*	CGC>TGC	R523C	w.t.	w.t.	w.t.	w.t.	>49	>53
Δ*mmp5-6*	CGC>CTC	R523L	w.t.	w.t.	w.t.	w.t.	>49	>53
Δ*mmp5-10*	w.t.	w.t.	TGA>CGA	Stop207R	w.t.	w.t.	8	4

## Supplementary Materials

The following are available online at https://www.mdpi.com/2079-6382/10/1/6/s1, Figure S1: Compound **1a**, ^1^H NMR (400 MHz) in CDCl_3_, Figure S2: Compound **1a**, HPLC, Figure S3: Compound **1b**, ^1^H NMR (500 MHz) in CDCl_3_, Figure S4: Compound **1b**, ^13^C NMR (126 MHz) in CDCl_3_, Figure S5: Compound **1b**, HPLC.
