# Supplementary material for: Identification of Mutations Conferring Tryptanthrin Resistance to Mycobacterium smegmatis
Source: Antibiotics (Basel). 2020 Dec 23;10(1):6. doi: 10.3390/antibiotics10010006 (PMC7823563; doi:10.3390/antibiotics10010006)

Figure S1: Compound 1a, ^{1}H NMR (400 MHz) in $CDCl_3$

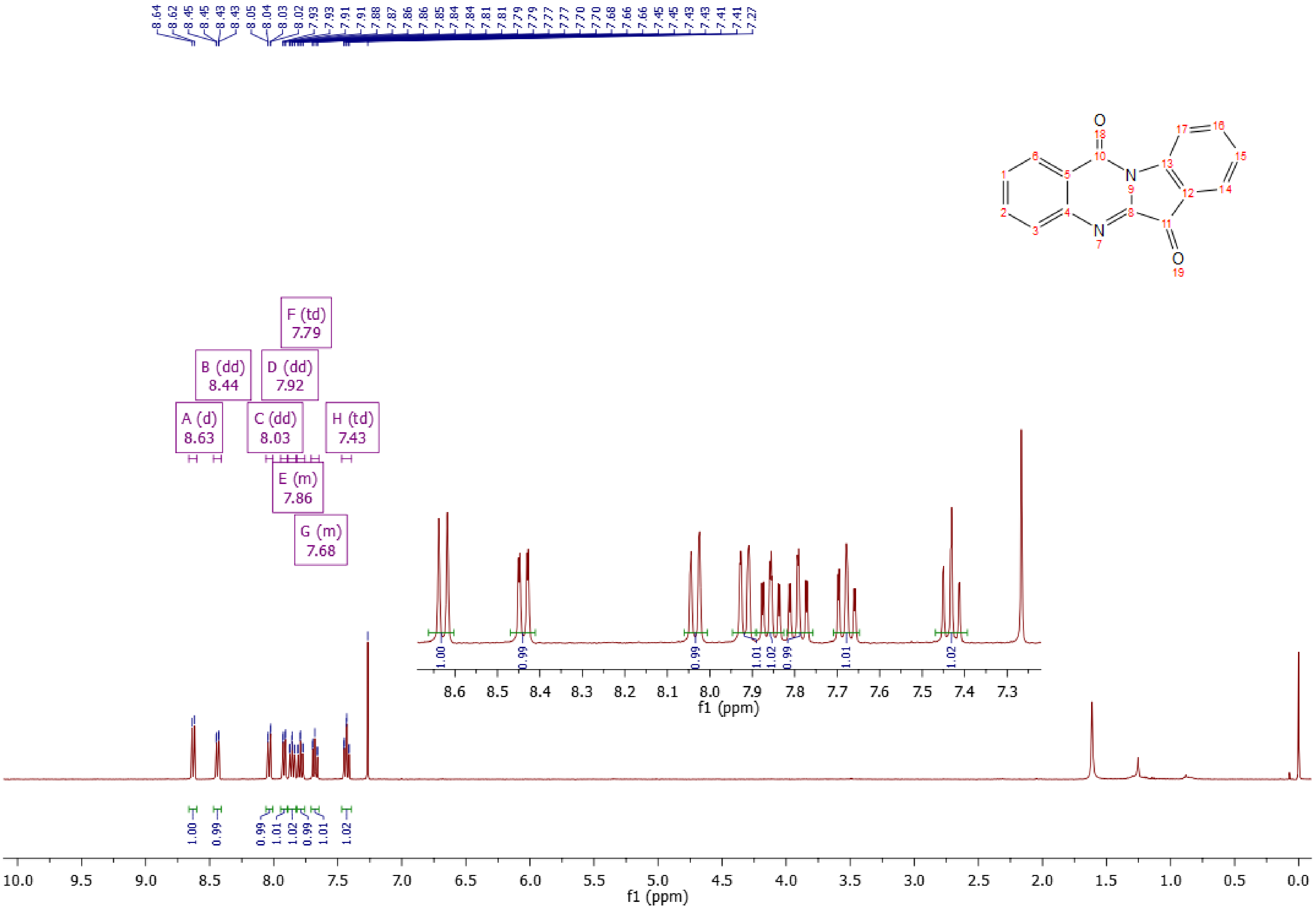

Figure S2: Compound 1a, HPLC

Panjab University Chandigarh

**Acquired By**	admin
**Sequence Name:**	SingleSample
**Sample name:**	RK-1-61
**Location:**	
**Inj. volume:**	
**Acq. method:**	Ravinder 1.amx
**Processing method:**	GC_LC Area Percent_DefaultMethod.pmx

Signal: VWD1A,Wavelength=250 nm

Name	RT	Area	Peak Area Percent	Peak Height	Peak Height Percent
	12.35	152.000	1.80	30.608	2.01
	14.41	8,308.475	98.20	1494.760	97.99

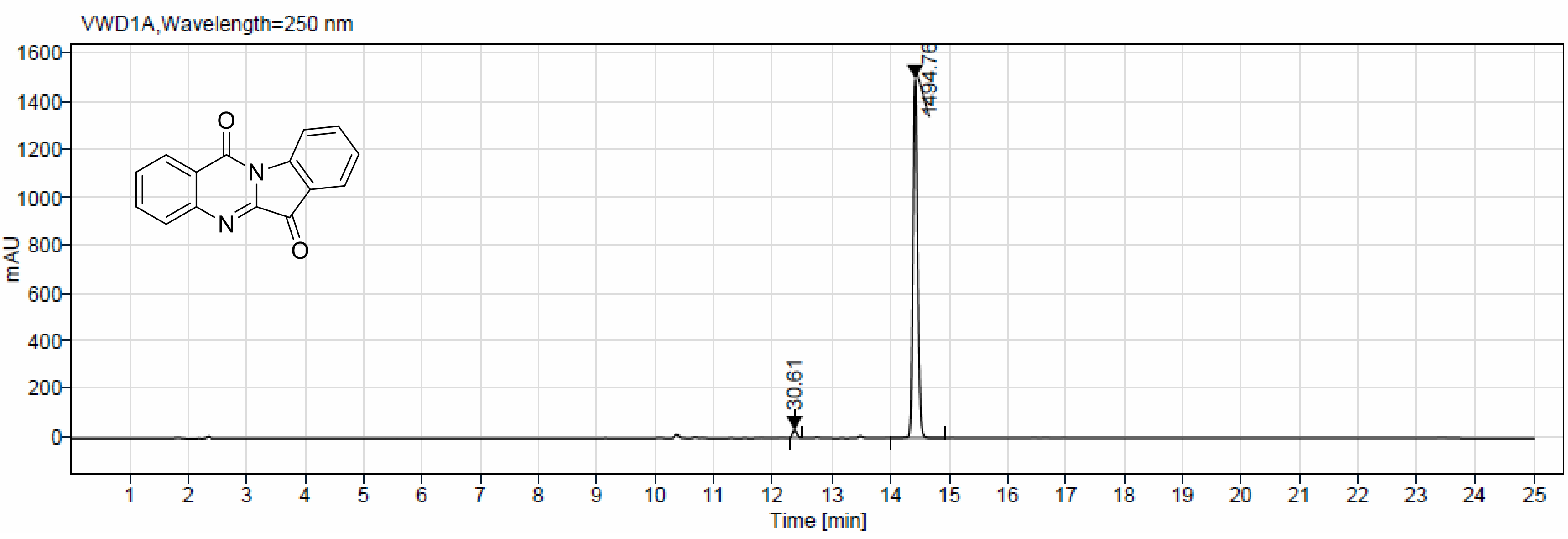

Figure S3: Compound 1b, ^{1}H NMR (500 MHz) in $CDCl_3$

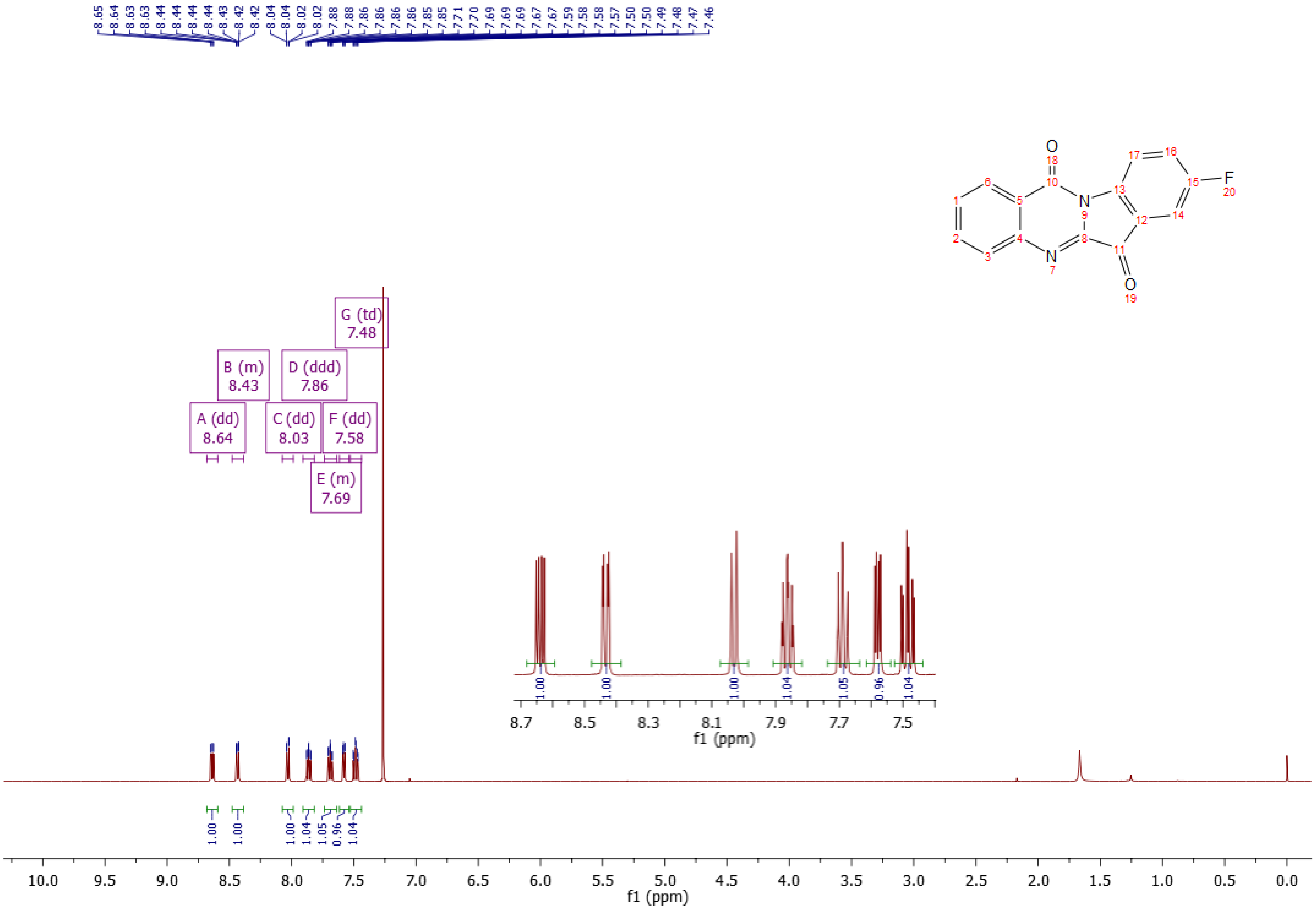

Figure S4: Compound 1b, ^{13}C NMR (126 MHz) in $CDCl_3$

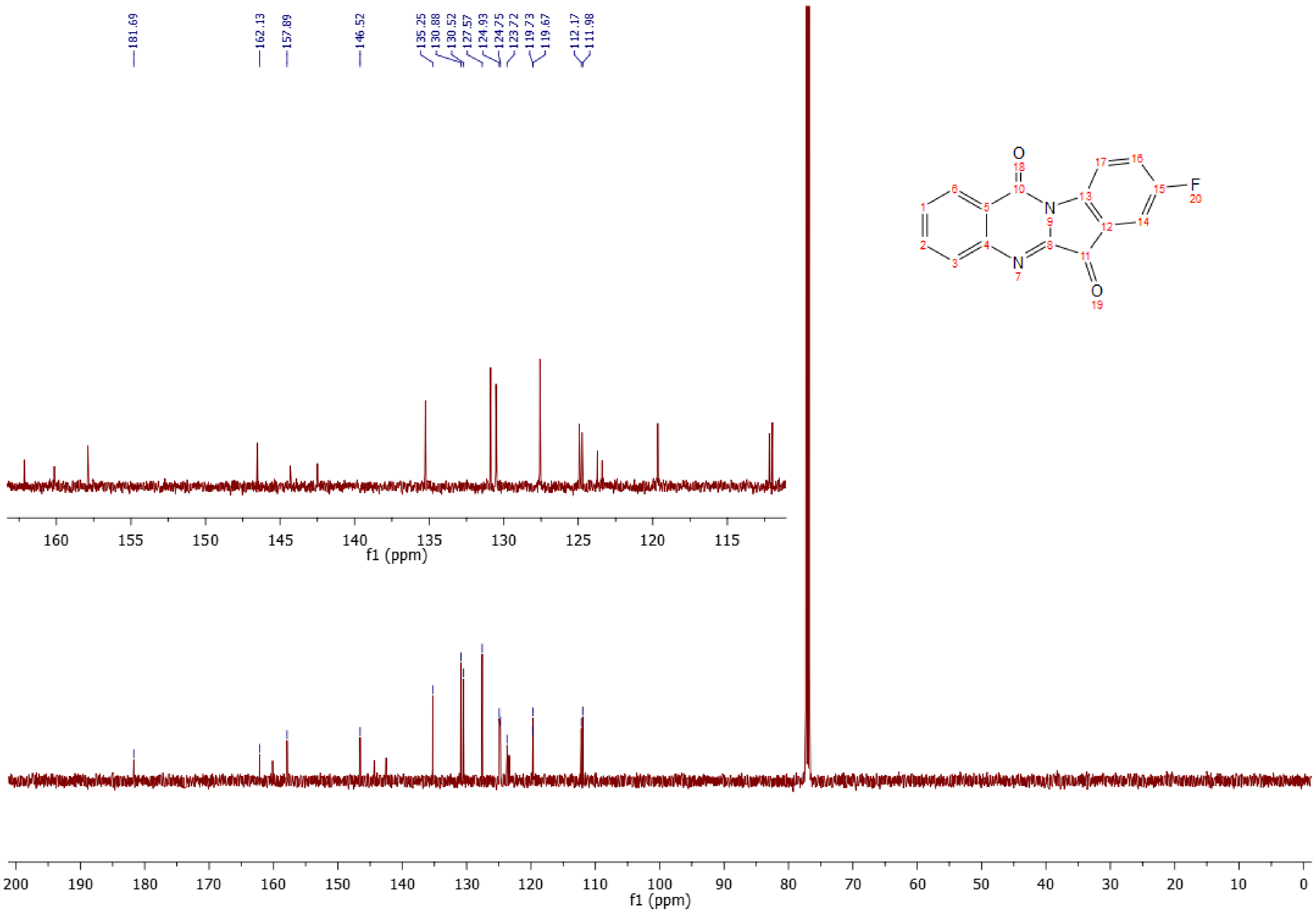

Figure S5: Compound 1b, HPLC

Panjab University Chandigarh

**Acquired By**	admin
**Sequence Name:**	SingleSample
**Sample name:**	RK-2-188
**Location:**	
**Inj. volume:**	
**Acq. method:**	Ravinder 1.amx
**Processing method:**	GC_LC Area Percent_DefaultMethod.pmx

Signal: VWD1A,Wavelength=250 nm

Name	RT	Area	Peak Area Percent	Peak Height	Peak Height Percent
	1.74	20.817	0.11	4.203	0.14
	1.80	24.565	0.13	4.467	0.15
	2.11	7.078	0.04	1.782	0.06
	2.38	30.191	0.16	7.745	0.26
	9.12	9.679	0.05	2.396	0.08
	11.01	61.877	0.32	9.302	0.31
	15.02	19,199.503	99.04	2942.547	98.86
	15.70	13.799	0.07	1.730	0.06
	16.41	18.822	0.10	2.401	0.08

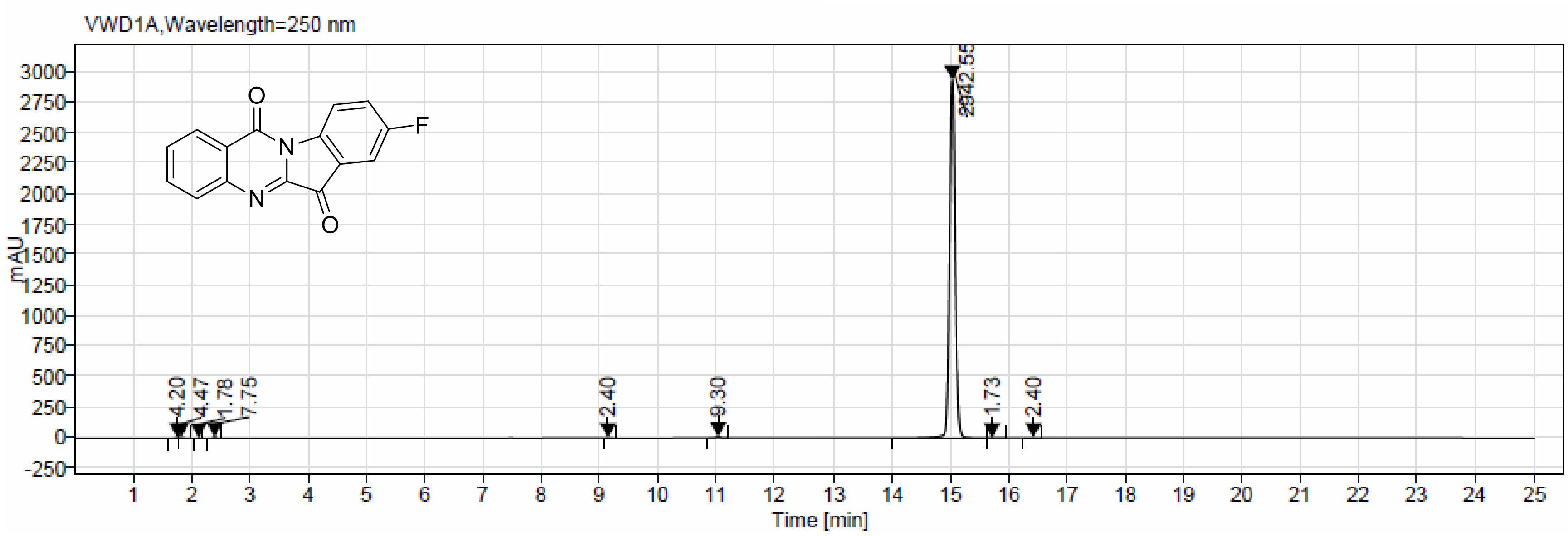

Supplement: Supplementary file 1 [file antibiotics-10-00006-s001.pdf]
